# ZnO Film Bulk Acoustic Resonator for the Kinetics Study of Human Blood Coagulation

**DOI:** 10.3390/s17051015

**Published:** 2017-05-03

**Authors:** Da Chen, Zhen Zhang, Jilong Ma, Wei Wang

**Affiliations:** State Key Laboratory of Mining Disaster Prevention and Control Co-founded by Shandong Province and the Ministry of Science and Technology, College of Electronics, Communications, and Physics, Shandong University of Science and Technology, Qingdao 266590, China; whuzhangz@163.com (Z.Z.); lxyjk@sdust.edu.cn (J.M.); skdwangwei1zqf@163.com (W.W.)

**Keywords:** ZnO film, film bulk acoustic resonator, viscosity sensor, coagulation monitoring

## Abstract

Miniaturized and rapid blood coagulation assay technologies are critical in many clinical settings. In this paper, we present a ZnO film bulk acoustic resonator for the kinetic analysis of human blood coagulation. The resonator operated in thickness shear resonance mode at 1.4 GHz. When the resonator contacted the liquid environment, the viscous loading effect was considered as the additional resistance and inductance in the equivalent circuits, resulting in a linear relationship with a slope of approximately −217 kHz/cP between the liquid viscosity and the frequency of the resonator. The downshift of the resonant frequency and the viscosity change during the blood coagulation were correlated to monitor the coagulation process. The sigmoidal trend was observed in the frequency response for the blood samples activated by thromboplastin and calcium ions. The coagulation kinetics involving sequential phases of steady reaction, growth and saturation were revealed through the time-dependent frequency profiles. The enzymatic cascade time, the coagulation rate, the coagulation time and the clot degree were provided by fitting the time-frequency curves. The prothrombin times were compared with the results measured by a standard coagulometer and show a good correlation. Thanks to the excellent potential of integration, miniaturization and the availability of direct digital signals, the film bulk acoustic resonator has promising application for both clinical and personal use coagulation testing technologies.

## 1. Introduction

Millions of patients use anticoagulant drugs to treat atrial fibrillation, pulmonary embolism, phlebothrombosis and other diseases. For these patients, it is very important to keep the blood rheological parameters on an appropriate level in daily life to avoid the risk of thrombus or bleeding. Currently, the widely-used coagulometers in the hospitals mainly are based on the detection of the change of blood viscosity during the coagulation process by paramagnetic particle methods or optical methods [1]. Although these technologies can provide quality-control and standardized results, they are an inconvenience for personal daily use because of the professional operation and expensive instrument. In addition, micromechanical resonators [2,3] and electroacoustic biosensors, such as the microcantilever [4], quartz crystal microbalance (QCM) [5,6,7] and surface acoustic wave (SAW) devices [8], have been used to characterize the kinetics of blood coagulation. The QCM sensor used for blood analysis is a kind of bulk acoustic wave (BAW) transducer composed of a piezoelectric plate and electrodes on one or two sides of the plate. An alternating electric field is applied on the electrodes to generate the shear mode acoustic resonance along the thickness direction. The resonant frequency of the BAW transducer is sensitive to the small mass addition on the upper surface or the change in the viscosity of adjacent mediums. QCM devices have been successfully used to monitor the blood coagulation in real time [9] and measure several hemostatic parameters, including activated partial thromboplastin time (aPTT) [10], prothrombinase induced clotting time (PiCT) [11], thrombin times (TTs) [12], prothrombin time (PT) [13] and fibrinogen [14]. This technology shows an outstanding application prospect in the point-of-care (POC) system. However, the typical QCM devices have a size of dozens of square millimeters and a frequency of several megahertz. Further miniaturization of QCM devices is limited by the capability of the traditional mechanical cutting and assembling method.

In the recent decade, based on micro-electromechanical system (MEMS) technologies, the film bulk acoustic resonator (FBAR) was developed as a promising micron-scale BAW sensor. FBAR devices employ 1–2 micron-thick piezoelectric ZnO [15,16], AlN [17,18] or ferroelectric films [19]. The working frequency at several GHz is significantly higher than that of the traditional QCM and improves the sensitivity substantially. Another advantage of FBAR is that this device is fabricated with MEMS technology, allowing batch fabrication, very small size and the integration in circuit chips. In recent years, FBAR devices have been successfully used as mass loading sensors for the detection of gas [20,21,22,23] and biological substances [24,25,26,27]. In these applications, the trace addition of the target material on the piezoelectric film causes a decrease in the resonant frequency [28]. On the other hand, as a BAW device, the resonant states of FBAR will be affected by the damping of adjacent medium when the device works in the liquid environment [29], which make it feasible to monitor the viscosity changes during the biological reaction in situ and in real time. However, the main challenge for the device is the excitation of the thickness shear mode resonance, capable of maintaining a good Q factor during in-liquid operation, because this mode hardly propagates in liquids, as opposed to the longitudinal mode. Effective excitation of such a mode requires the employment of electric fields perpendicular to the polarization axis of the piezoelectric film. For this purpose, many efforts have been made to grow inclined c-axis-oriented piezoelectric film coupled with two electrodes situated on the opposite sides of the film [30,31]. Besides, the use of lateral field excitation through coplanar electrodes in c-axis-oriented films has been proven to be an effective method [32,33].

In this paper, we fabricated a thickness shear mode ZnO FBAR using lateral field excitation for the study of coagulation kinetics of human whole blood. The frequency change of FBAR and the viscosity change during the blood coagulation were correlated. By following the frequency changes in the time domain, the sequential coagulation stages were revealed. The anticoagulant effect of heparin was demonstrated. Four clinically-significant parameters, including the enzymatic cascade time, the coagulation rate, the coagulation time and the clot degree were provided by fitting the time-frequency curves.

## 2. Device Configuration and Fabrication

### 2.1. The Structure of the FBAR Sensor

**Figure 1:**
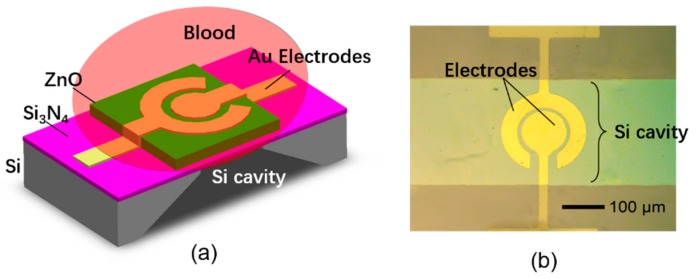
(**a**) The configuration of the film bulk acoustic resonator (FBAR)-based coagulation monitoring sensor; (**b**) the micrograph of the fabricated device.

Figure 1 shows the structure and micrograph of the FBAR-based coagulation monitoring sensor. The piezoelectric stack consists of a ZnO film (1 μm) and a pair of coplanar circular Au electrodes (120 nm/5 nm) with the gap of 10 μm. The thickness shear mode acoustic resonance at about 1.4 GHz was excited by the lateral electric field between the parallel electrodes. A polyethylene film (~120 nm) was deposited on the contacting surface to the blood sample to yield a hydrophilic surface that is favorable to fibrinogen absorption during the coagulation process. In addition, an air cavity was under the device to isolate the resonator acoustically from the silicon substrate.

### 2.2. The Device Fabrication Process

**Figure 2:**
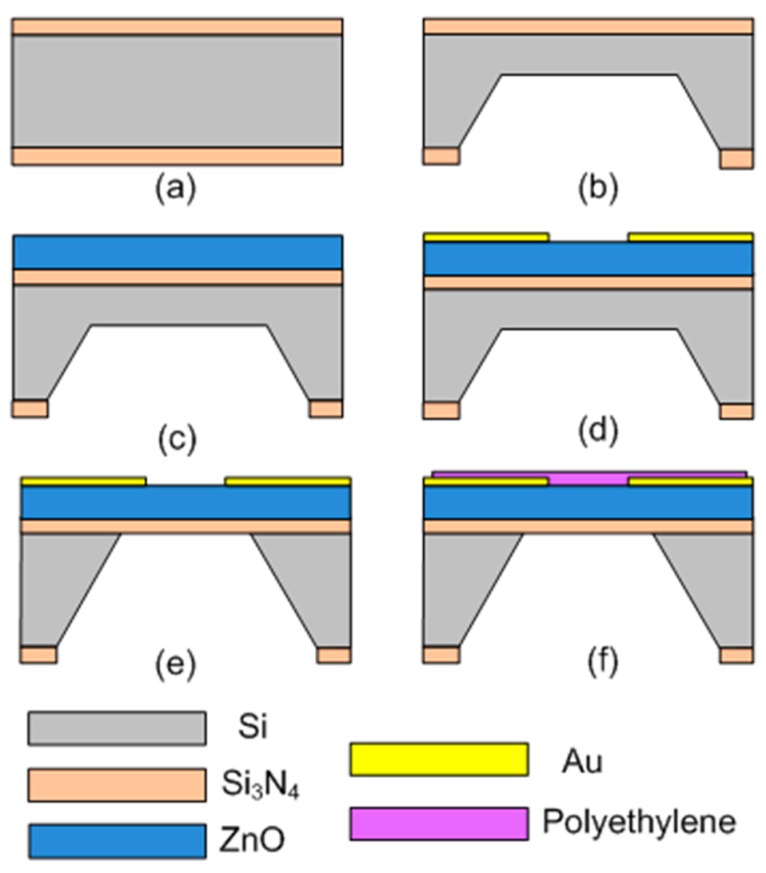
Fabrication process of the FBAR-based coagulation monitoring sensor. (**a**) Coating of the Si_3_N_4_ layer; (**b**) etching of the initial cavity; (**c**) deposition of ZnO film; (**d**) evaporation of electrodes; (**e**) releasing the piezoelectric stack; (**f**) spin-coating of the polyethylene layer.

As shown in Figure 2, the fabrication process of the FBAR device is described as follows. (a) The silicon (100) wafer was coated with low-stress Si_3_N_4_ layer (0.6 μm) on both sides of the wafer using plasma-enhanced chemical vapor deposition (PE-CVD, ZKY Co, LTD., Shenyang, China). (b) One side of the wafer was wet etched by hot KOH (80 °C) to form an initial cavity with the Si_3_N_4_ mask. About a 10 μm-thick silicon was left to support the following process. (c) The active ZnO film was deposited on the other side of the wafer using an RF magnetron sputtering system (JSD400, ZKY Co., LTD., Shenyang, China). The target was a four-inch-diameter ZnO with 99.99% purity. The process parameters were optimized to obtain a highly *c*-oriented ZnO film with good crystal quality (power: 2.5 kW; Ar: 30 sccm; O_2_: 20 sccm; chamber pressure: 1.5 Pa). (d) The Au electrodes were evaporated on the surface of the ZnO film and patterned by the standard lift-off process to generate the lateral electric field. (e) The residual silicon under the device was removed by deep reactive ion etching (DRIE) to release the piezoelectric stack from the substrate. (f) The resonator surface was spin-coated with 1 wt % polyethylene (Mw ~35,000, Sigma-Aldrich, Darmstadt, Germany) solution in decalin at 2500 rpm for 120 s and then incubated at 70 °C to evaporate the residual solvent.

### 2.3. The Circuit Diagram and Measurement Procedure

**Figure 3:**
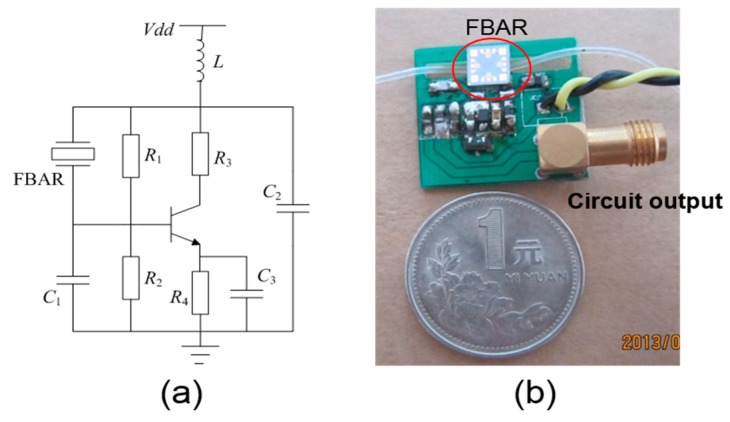
The circuit diagram (**a**) and the photography (**b**) of the assembled FBAR-based PCB for the blood coagulation monitoring.

Based on the fabricated FBAR device, an oscillator was assembled to realize a portable system for the real-time monitoring of whole blood coagulation. As shown in Figure 3, the FBAR device functions as an inductor in the oscillator circuit. The effective inductance of the resonator, *C*_1_ and *C*_2_, determines the oscillation frequency. The bipolar junction transistor (NE68119, Agilent, Santa Clara, CA, USA) is biased by *R*_1_, *R*_2_, *R*_3_ and *C*_3_. The oscillator output was connected to a network analyzer (Agilent 8714 ET, Agilent, Santa Clara, CA, USA) via a standard 50 Ω cable, and the data were processed by another computer.

The blood samples were collected from six healthy donors. All of the blood samples were treated with 3.8% sodium citrate solution (1:9) after the collection to prevent spontaneous clotting. The viscosity changes were detected in real time by following the frequency downshifts of the FBAR device during the blood coagulation. At first, the same volume of citrated blood sample and thromboplastin reagent (supplied by Guilin Urit Medical Equipment Co., LTD., Shanghai, China) were mixed uniformly in a centrifuge tube. Then, a droplet of 2 μL of the mixture solution was dripped on the resonator surface using a pipette. In order to initiate the blood coagulation, 1 μL 5–40 mM CaCl_2_ solutions were added into the liquid on the device surface. Four clinically-significant parameters, including the enzymatic cascade time, the coagulation rate, the prothrombin time (PT) and the clot degree, were provided by fitting the time-frequency curves. Here, the prothrombin time is defined as the end time of fibrin polymerization after the blood was activated by Ca^2+^ ions. Moreover, for the tests of anticoagulant sensitivity, the citrated blood samples were preincubated with the clinically-relevant concentration of unfractionated heparin (0–2 IU/mL) before adding the CaCl_2_ solution. Five times, measurements were performed for each concentrations. After each coagulation measurement, the device surface was scrubbed using 3% hydrogen peroxide solution and subsequently 10% oxalic acid solution to recover the original resonant frequency. In order to evaluate the accuracy of the proposed device, a commercial mechanical coagulometer (URIT-600, Guilin Urit Medical Equipment Co., LTD., Shanghai, China) was used to compare the PT values with the results measured by the FBAR device.

## 3. Results and Discussion

### 3.1. The Resonant Characterization of the FBAR Device

**Figure 4:**
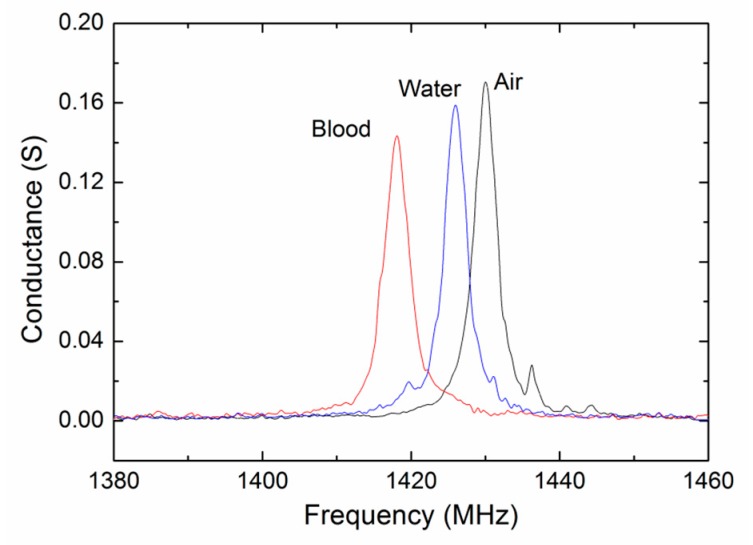
The conductance curves of the FBAR device measured in air, in pure water and in citrated blood at room temperature.

The resonant characterization of the shear mode ZnO FBAR device was firstly measured using the Cascade 9000 TM probe station with ACP 40 probes. Figure 4 shows the conductance curves (real part of the admittance) measured in air, in pure water and in citrated blood at room temperature. Clear shear mode resonances at about 1.4 GHz were observed in all of the situations. It is the shear mode resonance that is being excited because the corresponding acoustic velocity (~2900 m/s) is much closer to the theoretical value and other experimental values of the shear acoustic wave in the ZnO film. Compared with the case in air, slight decreases in the resonant frequency and declines in the conductance peak were found for the measurements in liquids.

**Figure 5:**
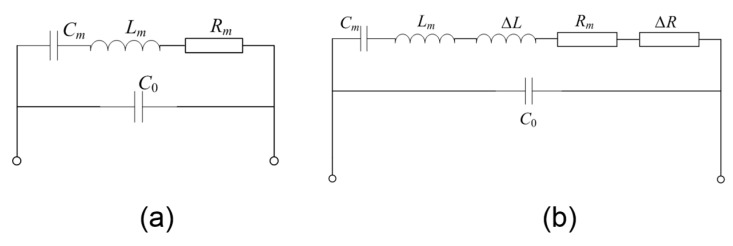
The Butterworth–Van Dyke (BVD) equivalent circuit of the FBAR device working in air (**a**) and in viscous liquid (**b**).

The electric characterization of FBARs are usually given by the Butterworth–Van Dyke (BVD) equivalent circuit [34,35] as shown in Figure 5a, where *C*_0_ is the clamped capacitance and *L*_m_, *C*_m_ and *R*_m_ are motional inductance, motional capacitance and motional resistance of the resonator, respectively. Table 1 summarizes the BVD parameters obtained by means of a least square fitting routine available in the Advanced Design System (ADS) software for the resonator working in air, in water and in blood, respectively. The resonant frequency (*f_s_* and *f_p_*) and Q factor are determined by the *LCR* parameters using the following:

(1) $$f_{s}=\frac{1}{2\pi \sqrt{L_{m}C_{m}}}$$

(2) $$f_{p}=f_{s}\sqrt{1+\frac{C_{m}}{C_{0}}}$$

(3) $$Q_{s/p}=\frac{2\pi f_{s/p}L_{m}}{R_{m}}$$

For the resonator working in a liquid environment, the viscous loading effect on the resonant performance can be considered as the additional resistance (Δ*R*) and inductance (Δ*L*) in the equivalent circuit as shown in Figure 5b. Obviously, the additional resistance and inductance results in the frequency downshift and a little attenuation in the *Q* factors.

**Figure 6:**
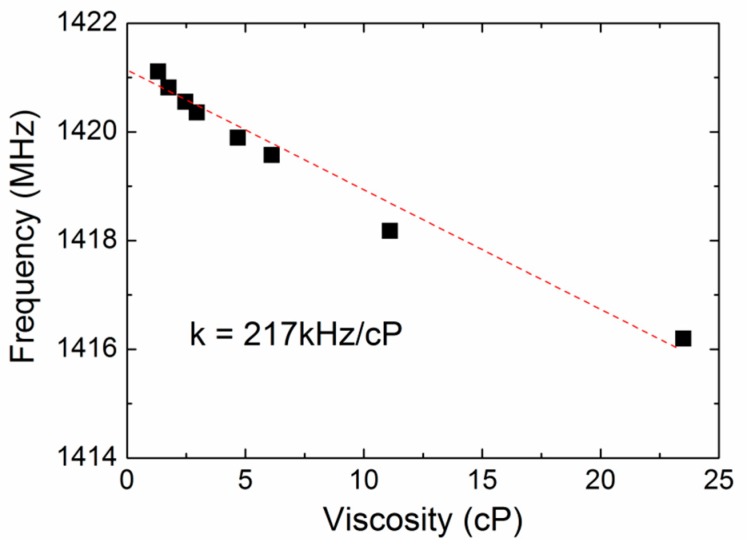
The resonant frequency of the FBAR device as a function of the liquid viscosity at room temperature.

The viscous response of the FBAR were then characterized using the aqueous glycerol solutions (0–70 wt %) with the viscosity of 1–23.5 cP. The aqueous glycerol solutions were chosen as the testing liquids to study the viscous effects on the proposed device because the density of the glycerol hardly varies with its concentration. Figure 6 shows the resonant frequency as a function of the liquid viscosity at room temperature. A linear relation with the slope of approximately −217 kHz/cP was observed between the frequency and viscosity. Based on this result, we can correlate the frequency change to the viscosity change of the target media by monitoring the resonant frequency change in the time domain.

### 3.2. The Monitoring of Blood Coagulation

**Figure 7:**
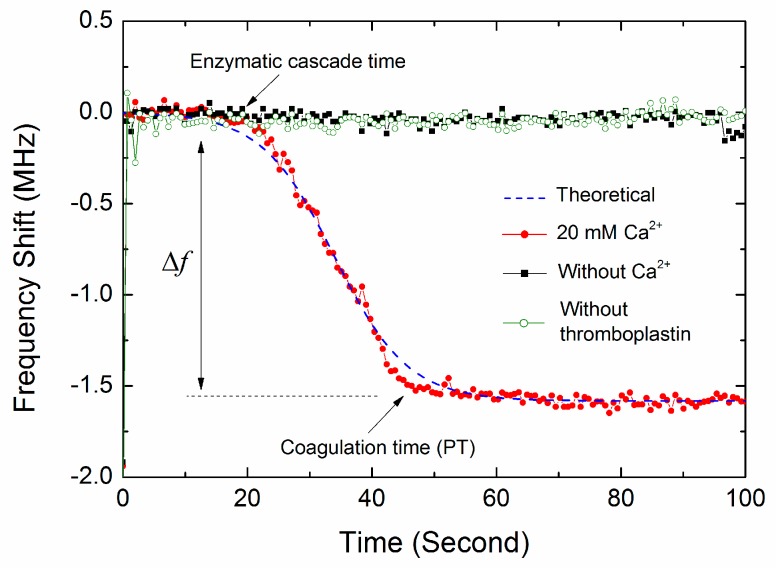
The representative real-time resonant frequency response of the FBAR device for the coagulation process. The coagulated blood sample was activated by thromboplastin and 20 mM CaCl_2_ solution.

Figure 7 shows a representative real-time resonant frequency response of the FBAR device for the coagulation process. The control measurement was performed using the same volume of pure water without the activators of thromboplastin or CaCl_2_ solution to exclude effects other than due to coagulation. There was an obvious difference between the blood coagulation curve and the control measurement curves. Without the activation of thromboplastin or Ca^2+^, the frequency nearly kept at a constant value, suggesting that there was no blood coagulation occurring. This constant frequency was given as the base line of the frequency shifts (Δ*f*) produced by coagulated blood samples. In the present of activators, the resonant frequency followed a sigmoidal trend involving the sequential phase of plateau, falling and saturation, revealing the different coagulation stages similar to those previously described [5,6,36]. Furthermore, as shown in Figure 7, the non-specific binding of the substances in blood (such as red/white cells, platelets, protein molecules) hardly has any influence on the resonator frequency over time in the observed region. In our experiments, the blood samples were first deposited on the resonator surface, and then, the Ca^2+^ ions were added to initiate the coagulation. It can be speculated that the non-specific binding has completed before the beginning of the coagulation process.

According to the extrinsic pathway of blood coagulation, the coagulation process mainly covers in turn the stages of cascade enzyme reaction, the fibrin formation and polymerization. The enzymatic cascade occurs first after the blood is activated by Ca^2+^ ions and thromboplastin. In this stage, thromboplastin (tissue factor) activates factor VII, which in turn leads to an activation of factor X. Together with activated factor V, Ca^2+^ ions and phospholipids-activated factor X form the prothrombinase complex. This complex catalyzes the conversion of prothrombin to thrombin. There are no obviously viscosity changes occurring in the first cascade enzyme reaction [37], resulting in the first plateau phase of resonant frequency. Then, thrombin as the central protein in the coagulation cascade cleaves fibrinogen to fibrin. The fibrin monomers polymerize into fibers, and the blood clot eventually forms. Consequently, the increase of liquid viscosity and the continuous formation of fibrin fibers result in that the resonant frequency gradually drops to a relatively low value and reaches a steady state at last, as shown in Figure 7. In the clinical applications, the end time of fibrin polymerization is determined as the prothrombin time.

It should be noted that other factors, such as the nonhomogeneous shear vibration, boundary effect and micro-form standing wave for the ultra-small sample volume, may influence the resonant state of the FBAR sensors [16]. Actually, the frequency shifts of the FBAR sensor were determined by the comprehensive effects during the blood coagulation instead of only the absolute viscosity change [5]. It is difficult to distinguish the contributions from the viscosity change and other factors in the frequency response. This is a limitation of the FBAR sensors for the application in the measurement of absolute blood viscosity. A detailed theoretical model to predict the effects of viscosity and other factors on the FBAR device will be studied in our further work.

### 3.3. The Coagulation Kinetics and Parameters

**Figure 8:**
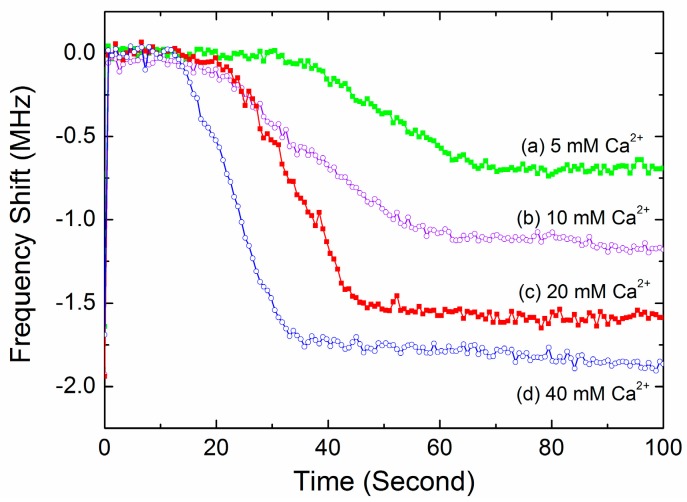
The typical time-dependent frequency profiles of the FBAR device for the blood samples activated by different Ca^2+^ ion concentration.

Figure 8 shows the typical time-dependent frequency profiles of the FBAR device for the blood samples activated by different Ca^2+^ ions concentration. The shapes of the sigmoidal curves were obviously dependent on the activation. Based on these observation, the time-dependent frequency profiles of the FBAR sensor were fitted using a sigmoid curve [38] as follows:

(4) $$f(t)=\frac{\Delta f}{1+e^{-k(t-t_{mid})}}$$

where *f(t)* is the frequency response at time *t*, *Δf* is the final frequency downshift, *k* is the reaction rate constant and *t_mid_* is the middle time at which the frequency equals the half value of *Δf*.

Four clinically-significant parameters, including the enzymatic cascade time, the coagulation rate, the PT and the clot degree, were derived from the real-time frequency response as summarized in Table 2. The final frequency downshift is associated with the density of the produced fibrin network and can be considered as a measure of clot degree. As expected, the blood sample activated by higher Ca^2+^ concentration has both higher coagulation rate *k* and larger frequency downshift Δ*f*. The times when the frequency decreased to three typical percentages (90%, 95%, 100%) of the final frequency downshift (Δ*f*) were compared with the results acquired by the commercial coagulometer. The results showed that the times when the frequency decreased to 95% have the best correlation to the coagulometer (see Section 3.5). Consequently, these characteristic points were determined as the PT values of the blood samples. In addition, for all of the blood samples, the coefficient of variation (CV) of the coagulation parameters (Table 2 and Table 3, Supplementary Materials Tables S1 and S2) could be seen to be low enough to show reproducibility, considering the normally accepted 10% level for a reasonable analytical method.

All of the data were calculated based on the average values of the blood samples collected from six donors and five measurement times for each condition.

### 3.4. Test of the Inhibiting Effect of Heparin

**Figure 9:**
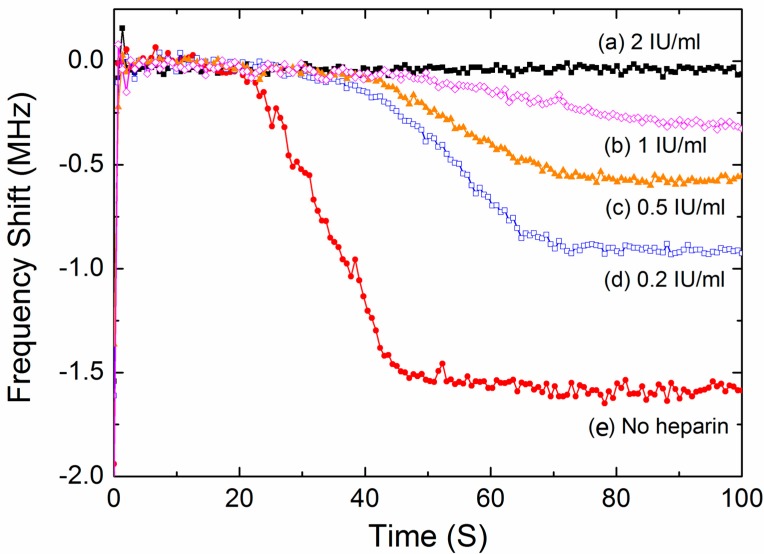
The typical time-dependent frequency profiles of the FBAR device for the blood samples added with different concentrations of heparin.

In clinical practice, heparin is a typical anticoagulant drug to treat and prevent blood clots. Heparin binding to thrombin alters the affinity towards fibrinogens, which inhibits the fibrin formation and thus slows down the coagulation process. In this work, the FBAR sensor was used to demonstrate the anticoagulant effect by adding heparin to the blood samples. Figure 9 shows the representative time-dependent resonant frequency profiles for different heparin concentrations. The shapes of the frequency profile were closely dependent on the heparin concentration. In particular, when an excess quantity of anticoagulant was added in the blood sample, no coagulation process was observed during the test time. Based on the fitting Equation (4), the coagulation parameters were obtained from the frequency response as summarized in Table 3. Obviously, the presence of heparin results in lower reaction rates and smaller frequency downshifts because of the inhibiting effect of heparin on the blood coagulation.

All of the data were calculated based on the average values of the blood samples collected from six donors and five measurement times for each condition. All of the blood samples were activated by 20 mM CaCl_2_ solution.

### 3.5. Comparison between the FBAR Device and Standard Coagulometer

**Figure 10:**
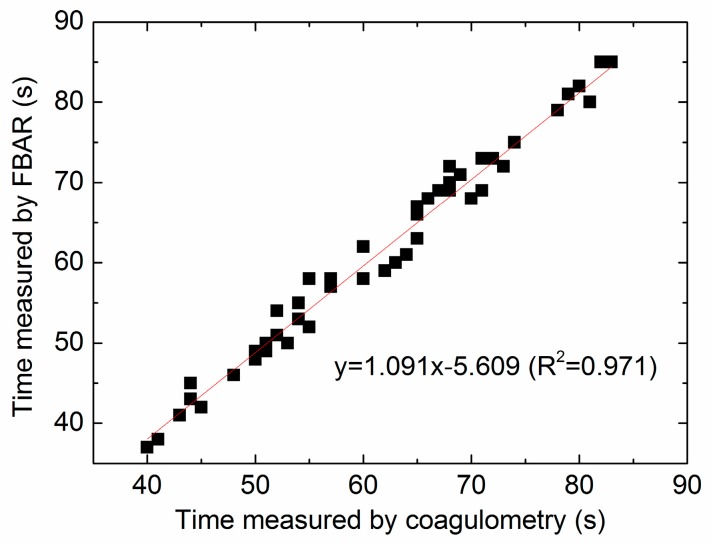
The correlation between the PT values determined by the FBAR sensor and the results acquired by the commercial coagulometric method.

In order to validate this new measurement technique, the blood samples from six healthy donors were studied. Figure 10 shows the correlation between the PT values determined by FBAR sensor and the results acquired by the commercial coagulometric method. The blood samples were activated by different concentration CaCl_2_ or added with different concentrations of heparin. Importantly, in all of the coagulation time analyses done in this study, it is found that the correlation (*R*^2^) of the two methods was 0.971, where *R*^2^ = 0 indicates that the FBAR sensor is fully inaccurate, and *R*^2^ = 1 represents a perfect measurement. Therefore, the coagulation time determined from the time-frequency profiles of the FBAR sensor (corresponding to 95% of Δ*f*) can act as a reliable quantitative end point of the coagulation assay. In comparison, the correlation values corresponding to 90% and 100% characteristic points are 0.913 and 0.892, respectively.

The major feature of the blood coagulation monitoring device described here is that it can be batch-manufactured at the wafer level at low cost utilizing the existing MEMS process. The FBAR sensors have the feasibility to be arrayed and integrated with the CMOS electronics in a single-chip configuration [39]. Furthermore, the miniaturization of the MEMS-based testing system also facilitates reducing the specimen consumption. This means that each blood test requires a smaller pinprick and hurts less. In this work, the FBAR sensor has a very small size within 0.1 × 0.1 mm^2^, and the sample consumption is only 2 μL, which is much less than that of the existing coagulometer, such as the thrombelastogram, QCM, the turbidimetric and mechanical meter. In addition, the FBAR sensors are small and light enough to be placed at a high density within the microfluidic channels by MEMS technologies. For the clinic applications in personal daily management of anticoagulant drugs, future efforts should be devoted to develop a POC system that combines the FBAR sensor array and the microfluidic blood handling components. Blood coagulation assays can be run automatically using extremely small sample volumes, making the existing laboratory-based coagulation diagnostics accessible at home.

## 4. Conclusions

We proved that the shear mode ZnO FBAR-based viscosity sensor was able to monitor the human blood coagulation process in real time. The resonant frequency of the FBAR sensor decreased along a sigmoid curve during the blood coagulation process. The shapes of the time-dependent frequency profiles were obviously dependent on the activation of Ca^2+^ ions. Based on the curve-fitting results, the sequential coagulation stages and four coagulation parameters were studied. The inhibiting effect of heparin on the blood coagulation were obvious. The measured prothrombin time shows a good linear correlation and consistency with the results obtained from a commercial coagulometer. The proposed FBAR sensor, which has the advantages of small size, light weight, low cost, simple operation and low sample consumption, is a promising device for miniaturized, online and automated analytical system for routine diagnostics of hemostatic status and personal health monitoring.

## Figures and Tables

**Table 1:** The BVD parameters of the FBAR device working in air, in water and in blood.

Parameters	In Air	In Water	In Blood
*f_s_* (GHz)	1.430	1.426	1.418
*f_p_* (GHz)	1.441	1.437	1.429
*Q_s_*	430	404	376
*Q_p_*	433	407	379
*C*_0_ (pF)	2.73	2.69	2.63
*C_m_* (fF)	44.8	44.2	43.3
*L_m_* (μH)	27.6	28.2	30.4
*R_m_* (Ω)	5.78	6.21	7.22

**Table 2:** The clinic parameters derived from the real-time frequency response for the blood samples activated by different Ca^2+^ concentrations. PT, prothrombin time.

Ca^2+^ Concentrations (mM)	Enzymatic Cascade Time (s)	Coagulation Rate Constant *k*	Finial Frequency Downshift Δ*f* (MHz)	PT (s)	CV of PT
5	30 ± 2.0	0.13 ± 0.006	0.79 ± 0.06	64 ± 5.5	8.5%
10	23 ± 1.8	0.17 ± 0.021	1.21 ± 0.08	58 ± 4.8	8.2%
20	18 ± 1.1	0.18 ± 0.012	1.63 ± 0.09	52 ± 3.0	5.7%
40	15 ± 2.0	0.22 ± 0.020	1.92 ± 0.15	42 ± 2.4	5.7%

**Table 3:** The clinic parameters derived from the real-time frequency response for the blood samples added with different concentrations of heparin.

Heparin Concentrations (IU/mL)	Enzymatic Cascade Time (s)	Coagulation Rate Constant *k*	Finial Frequency Downshift Δ*f* (MHz)	PT (s)	CV of PT
0	18 ± 1.1	0.18 ± 0.012	1.63 ± 0.09	52 ± 3.0	5.7%
0.2	35 ± 2.4	0.15 ± 0.012	0.91 ± 0.04	70 ± 4.0	5.7%
0.5	41 ± 3.6	0.12 ± 0.008	0.58 ± 0.02	72 ± 4.3	6.0%
1	46 ± 3.8	0.08 ± 0.004	0.29 ± 0.04	80 ± 7.4	9.3%

## Supplementary Materials

The supplementary materials are available online at http://www.mdpi.com/1424-8220/17/5/1015/s1.
