# Supplementary material for: ZnO Film Bulk Acoustic Resonator for the Kinetics Study of Human Blood Coagulation
Source: Sensors (Basel). 2017 May 3;17(5):1015. doi: 10.3390/s17051015 (PMC5469538; doi:10.3390/s17051015)
Supplement: Supplementary file 1 [file sensors-17-01015-s001.pdf]

Table S1 The C.V. values of the coagulation parameters for the blood samples activated by different Ca^{2+} concentrations.

**Ca^{2+} concentrations (mM)**	**Enzymatic cascade time**	**Coagulation rate constant k**	**Finial frequency downshift Δf**	**PT**
5	6.7%	4.6%	7.5%	8.5%
10	7.8%	12.3%	6.6%	8.2%
20	6.1%	6.7%	5.5%	5.7%
40	13.3%	9.1%	7.8%	5.7%

Table S2 The C.V. values of the coagulation parameters for the blood samples added different concentration heparin.

**Heparin concentrations (IU/ml)**	**Enzymatic cascade time**	**Coagulation rate constant k**	**Finial frequency downshift Δf**	**PT**
0	6.7%	4.6%	7.5%	8.5%
0.2	6.8%	8.0%	4.3%	5.7%
0.5	8.7%	6.7%	3.4%	6.0%
1	8.2%	5.0%	13%	9.3%
